# Automated Detection of the Kyphosis Angle Using a Deep Learning Approach: A Cross-Sectional Study on Young Adults

**DOI:** 10.3390/diagnostics15111422

**Published:** 2025-06-03

**Authors:** Onur Kocak, Cansel Ficici, Ilknur Ezgi Dogan, Ziya Telatar, Nihan Ozunlu Pekyavas

**Affiliations:** 1 Department of Biomedical Engineering, Faculty of Engineering, Başkent University, Ankara 06790, Turkey; ztelatar@baskent.edu.tr; 2 Department of Electrical and Electronics Engineering, Faculty of Engineering, Ankara University, Ankara 06830, Turkey; ogretmenoglu@ankara.edu.tr; 3 Department of Physiotherapy and Rehabilitation, Başkent University, Ankara 06790, Turkey; ezgidogan@baskent.edu.tr (I.E.D.); nozunlu@baskent.edu.tr (N.O.P.)

**Keywords:** automated posture analysis, deep learning, kyphosis angle, medical decision support system

## Abstract

**Objectives:** In healthy young adults, thoracic kyphosis can be attributed to a number of factors, including a sedentary lifestyle, stress, poor posture, activity and daily habits, muscle pain, fatigue, and anxiety. In regard to clinical diagnosis and evaluation methods, high-cost radiological measurements and a variety of non-radiological clinical methods are employed. In this study, a decision support system that performs automatic thoracic kyphosis angle measurements has been developed with the objective of avoiding exposure of the human body to radiation and reducing the time required for measurements. **Methods:** The features were determined with reference to the thoracic kyphosis measurements that were manually marked by the expert on the subjects. The kyphosis angle was calculated by automatically identifying the T1 and T12 points through image segmentation using a convolutional neural network (CNN), which is a type of deep learning algorithm. **Results:** Intra-class consistency of ICC > 0.95 (*p* < 0.05) and internal consistency reliability of Cronbach’s α = 0.947 are obtained. **Conclusions:** The results demonstrate that the proposed algorithm exhibits high intra-class consistency and high internal consistency reliability to provide an automated thoracic kyphosis angle measurement system.

## 1. Introduction

Thoracic kyphosis is a natural, physiological curvature formed by the vertebrae and discs in the form of an anterior concave between the T1 and T12 vertebrae in the sagittal plane. While thoracic kyphosis is considered “normal” at values between 20° and 40° in young adults [1], it has been observed that increased kyphosis can be seen in adults older than 40 years of age. In older adults, age-related increased kyphosis can reach up to 50° [2,3].

Postural thoracic kyphosis, defined between 20 and 40 degrees, describes increased kyphotic curvatures resulting from postural habits, irrespective of genetics or the impact of various diseases that affect the vertebral column structurally. In examining the factors that contribute to postural kyphosis, activities that promote poor posture, such as a sedentary lifestyle, limited mobility, weak postural muscles, and prolonged use of technological devices, warrant particular attention. Consequently, in addition to the deterioration in the individual’s quality of life, musculoskeletal issues and pain are frequently encountered as a consequence of increased thoracic kyphosis. A study has indicated that an increase in thoracic kyphosis results in a disruption to the scapular rhythm, leading to a reduction in shoulder movement in multiple directions [4]. Furthermore, it has been documented that an excessive increase in thoracic kyphosis has a detrimental impact on shoulder joint mobility and is a primary cause of cervical pain [5]. Furthermore, it has been documented that as thoracic kyphosis progresses, there is a concomitant weakening of the respiratory system and an increase in pulmonary problems. This is attributed to factors such as increased abdominal pressure and the pressure of the ribs on the diaphragm [6]. Consequently, while increased thoracic kyphosis has a detrimental impact on the individual’s quality of life, it necessitates treatment due to the emergence of secondary complications and results in elevated healthcare expenditures. In such cases, it is crucial to accurately assess the degree and severity of the curvature through the use of valid and reliable evaluation methods and to intervene early.

The method most widely accepted as the gold standard for evaluating thoracic kyphosis is the Cobb angle, which is obtained by lateral radiographic measurement [7]. The Cobb angle is a valid and reliable method frequently used in diagnosis [8]. Although radiographic evaluation provides highly reliable results regarding the degree of thoracic curvature, it has been asserted that it exposes the patient to elevated levels of potentially harmful radiation, is not readily accessible in a clinical setting, and its utilization is constrained due to its cost [4]. In light of these considerations, a range of alternative methods have been developed for the clinical evaluation of thoracic kyphosis, which may offer a viable alternative to radiographic assessment. The flexicurve meter is particularly suited to widespread use in the clinic, offering a simple, low-cost solution. However, although the measurement process with a flexicurve meter is relatively straightforward, it necessitates the performance of calculations on millimetric paper, and the experience of the evaluator may potentially influence the results obtained. Furthermore, it has been demonstrated that there is a considerable correlation between the Cobb angle and the data obtained from clinical measurement tools [1,2,4]. It is important to note that the choice of measurement tool may be influenced by various factors, including the strength of the paravertebral muscles in a standing position or the stretching effect on the thoracic region depending on the evaluation position. This highlights the necessity for a more sensitive measurement tool to ensure accurate and reliable results.

A substantial number of studies in the literature have employed manual measurement of the Cobb angle or kyphosis. In a study published in 2020, Grindle [9] and colleagues investigated a method for estimating the kyphosis angle that combined the flexicurve, Cobb angle-based marker, and camera systems. The authors compared their evaluation method with three different non-radiological and commonly used estimation methods, finding disparate values and weak to moderate correlations with each of them.

The use of convolutional neural networks (CNNs) provides some advantages in automatically extracting hierarchical spatial features from complex visual data. In addition, CNN models show significant advantages in segmentation tasks. Specifically, CNN-based models can accurately identify object boundaries and capture fine-grained structural details, which are essential for achieving high segmentation precision.

Due to all these necessities, the aim of this study is to develop an assessment tool based on a deep learning approach that provides automatic measurement of kyphosis angles and to determine its validity in young adults.

The following is a description of the structure of the article: In Section 2, the dataset utilized in this study is presented. Furthermore, the proposed algorithm is elucidated in comprehensive detail. Section 3 presents the results of the deep learning algorithm and kyphosis angle detection method. Section 4 presents the conclusions, contributions, and novel aspects of the paper.

## 2. Materials and Methods

In this study, images of 125 subjects were captured in their everyday attire. The T1 and T12 points, as identified by the expert, and the features derived from the training set, along with the deep learning architecture, were trained for segmentation. Initially, the silhouettes of the subjects within the images were segmented using a CNN algorithm.

Subsequently, the T1 and T12 points of the subjects were automatically detected in the test images by leveraging the features acquired from the training set. Subsequently, the kyphosis angle was calculated automatically using the coordinates of the T1 and T12 points.

### 2.1. Subjects and Demographics

Healthy adults between the ages of 18 and 40 were included in the study with the approval of the Başkent University Non-Interventional Clinical Research Ethics Committee (approval number: E-91694447-604.01-345559, dated 22 May 2024). Written informed consent was obtained from all participants. Volunteers who met the inclusion criteria were enrolled in the study. Clinical evaluations were conducted by a physiotherapist who is an expert in the field with 12 years of professional experience. The inclusion criteria were as follows: being between 18 and 40 years of age and volunteering to participate in the study. Exclusion criteria included: having a diagnosed rheumatic, systemic, or neurological disease affecting the musculoskeletal system (particularly the spine); having a diagnosis of scoliosis; having undergone spinal surgery; and being pregnant.

### 2.2. Image Collection and Dataset Augmentation

In this study, the Intel^®^ RealSense™ Depth Camera (D435i; Intel Corporation, Santa Clara, CA, USA) was used for non-radiographic imaging. It provides a maximum depth resolution of 1280 × 720 pixels and a RGB resolution of up to 1920 × 1080 pixels. The system supports depth streaming at up to 90 frames per second (FPS) and offers a diagonal field of view of approximately 70°. The effective depth sensing range varies from 0.3 m to over 4 m. The physical dimensions of the camera are approximately 90 mm × 25 mm × 25 mm.

In this study, a dataset was constructed by taking images of subjects who had provided voluntary consent to participate. The images of the subjects were captured at consistent distances and with an identical background using the camera system installed in the university laboratory. The dataset comprises 125 sagittal images of subjects in a standing position, with the dorsal region visible.

**Figure 1:**
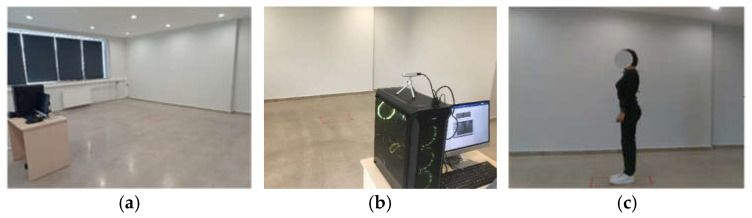
(**a**) University laboratory, (**b**) installed camera system, (**c**) participant in the evaluation area.

Research Figure 1 depicts the camera system installed in the university laboratory and a participant engaged in the evaluation process. The dataset was augmented through the application of a data augmentation technique. As a result of the background modification, the dataset was expanded to comprise 375 images.

### 2.3. Clinical Assessment with Flexicurve Meter

Following the recording of the participants’ demographic information, the evaluation of their thoracic kyphosis was conducted with the flexicurve system. Thereafter, the data obtained from the evaluations were calculated and subjected to statistical analysis. Furthermore, the camera system utilized in the university laboratory enabled the acquisition of images of the subjects at identical distances and with an identical background, thus facilitating the creation of a comprehensive dataset.

The reliability of non-radiological measurement methods used to assess thoracic kyphosis is comparable to that of the Cobb angle [10]. Among these non-radiological measurement methods, the Debrunner measurement method, the kyphosis angle, the flexicurve kyphosis index, and the flexicurve are the most commonly utilized in clinical settings. The findings of the study indicate that there is no discernible difference between these methods in terms of the degree of concordance between their evaluation results for thoracic kyphosis and those obtained using the Cobb angle. In the clinical evaluation of thoracic kyphosis, the flexicurve meter was employed. The measurement and calculation procedures of the assessment with the flexicurve tool took 15 min for each participant.

**Figure 2:**
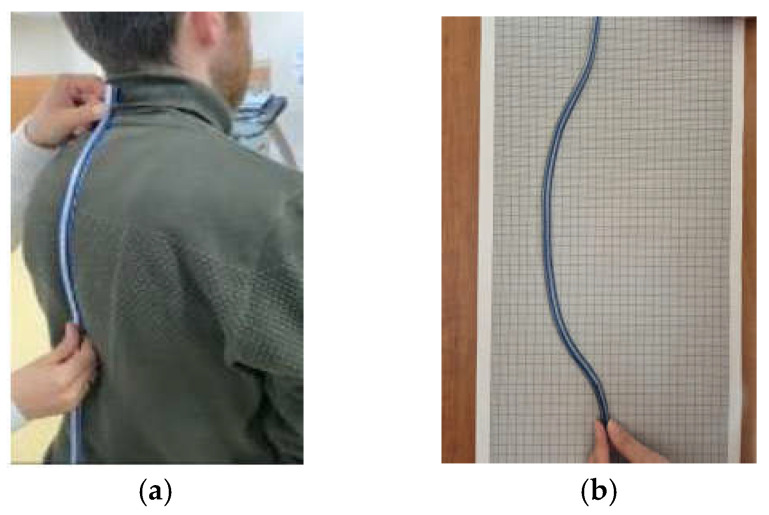
(**a**) Measuring thoracic curvature with flexicurve. (**b**) Transferring the flexicurve meter to millimetric paper.

The subject was instructed to assume an upright, comfortable posture without any clothing. The thoracic curvatures of the participants were measured by a physiotherapist from the thoracic spine between T1 and T12 in full contact with the flexicurve meter (Figure 2a). The data obtained from the flexicurve meter were transferred to the millimetric paper surface (Figure 2b), and the depth (*d*) and length (*L*) values were determined and recorded in the evaluation form [9] (Figure 2). The *d* and *L* data obtained from this measurement were calculated using the formula given in (1) and recorded as theta value for use in the statistical analysis of the participants whose clinical measurement was completed [9].

(1) $$\theta =2\sin^{-1}\left(\frac{4dL}{4d^{2}+L^{2}}\right)$$

### 2.4. Automated Kyphosis Angle Detection Algorithm Using CNN

**Figure 3:**
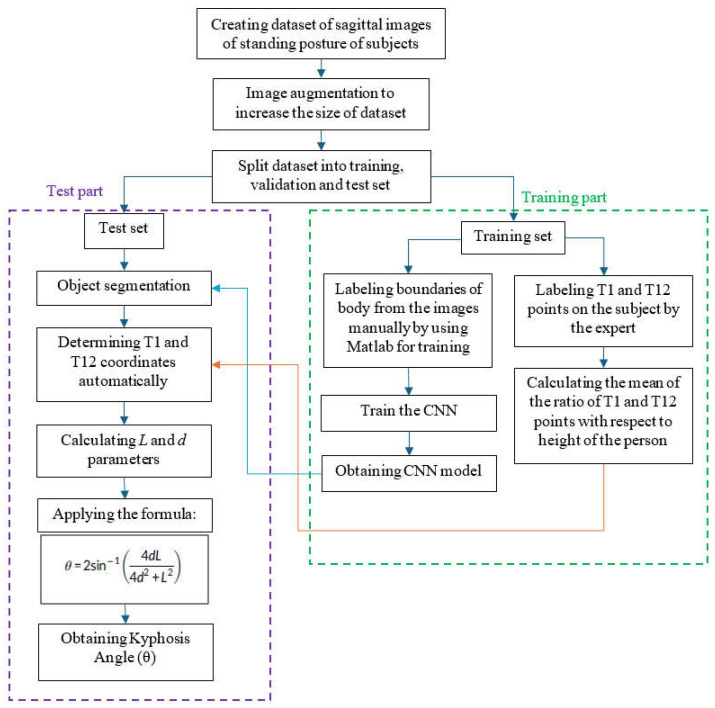
Flowchart of the proposed automated kyphosis angle detection algorithm.

The proposed algorithm was implemented with MATLAB 2021a [11] via a computer with an Intel Core i7 processor (2.60 GHz; Intel Corporation, Santa Clara, CA, USA) and 16.0 GB RAM. The flowchart of the proposed algorithm is provided in Figure 3 for reference.

The initial step involved the creation of the dataset, which was obtained by capturing the participants in a rectangular area three meters away from the camera system installed in the university laboratory, with the participants standing in front of an empty background. The dataset comprises sagittal images of 125 subjects, with the dorsal region visible. To augment the dataset, the number of images was increased to 375 by modifying the backgrounds of each image. Furthermore, publicly available segmentation images [12] were incorporated into the existing dataset to enhance the performance of the CNN algorithm in terms of segmentation. The publicly available dataset comprises images with manually labeled person instances. In conclusion, the dataset was augmented to comprise 553 images, which were included in the study. The publicly available segmentation images were utilized exclusively for training purposes and were not employed in the testing phase. The 375 images in the dataset have been divided into three sets for the purposes of training, validation, and testing, with 70%, 15%, and 15% of the images, respectively, allocated to each set. Subsequently, the body boundaries of each subject were delineated manually via the MATLAB segmentation application, thus constituting the training stage of the CNN algorithm. Once the training and labeled data had been obtained, the training process of the CNN algorithm was initiated. The CNN algorithm employs the ResNet50 structure. The maximum number of epochs, mini-batch size, and initial learning rate were set to 10, 4, and 0.001, respectively. The training phase yielded the results required to generate the CNN model to be used in semantic segmentation.

The training phase of the automated semantic segmentation algorithm with CNN was completed in 19 min and 20 s, while the test duration for each subject was 0.81 s.

**Figure 4:**
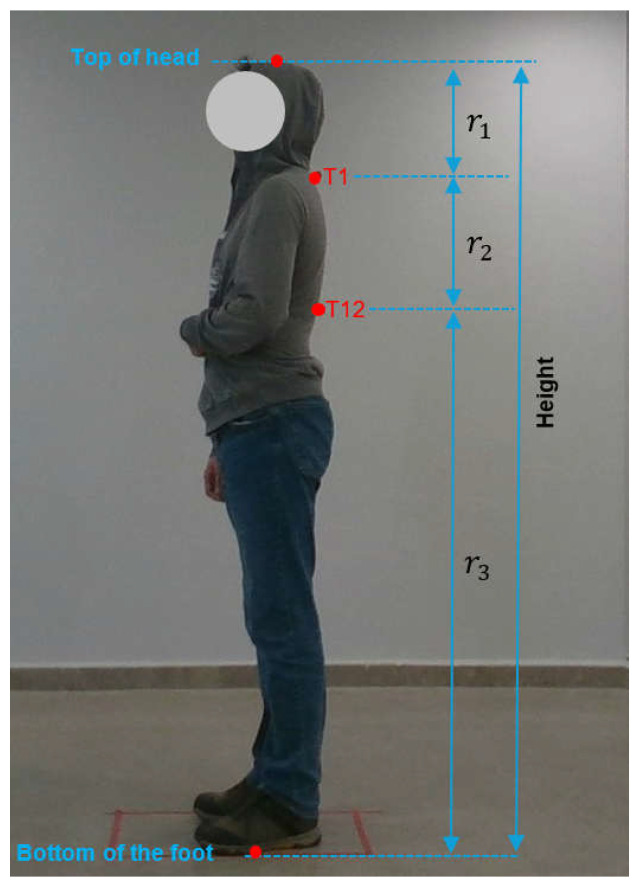
Marking certain points to calculate $r_{1}$, $r_{2}$, and $r_{3}$.

The coordinates of the T1 and T12 points identified by the subject matter expert (Figure 4) through manual measurement were determined on the image. Furthermore, the coordinates of the cranial vertex and the points of ground contact were also determined on the image. The ratios of the distances to T1 and T12 points were determined for each subject with reference to the coordinates of the head and foot. The ratios $r_{1}$, $r_{2}$, and $r_{3}$ were subsequently calculated by averaging the ratios for all subjects. As illustrated in Figure 4, the ratio of the distance between the top of the head and T1 to the height of the person (in pixels) was designated as $r_{1}$, the ratio of the distance between T1 and T12 to the height of the person was designated as $r_{2}$, and the ratio of the distance between T12 and the bottom of the foot to the height of the person was designated as $r_{3}.$ By employing the Equations between (2) and (5), the height of a training subject, along with the average ratios $r_{1}$, $r_{2}$, and $r_{3}$, can be calculated. The pixel coordinates of the top of the head, T1 point, T12 point, and the bottom of the foot of the *i*th training subject are represented by $P_{i1}=(x_{i1},y_{i1})$, $P_{i2}=\left(x_{i2},y_{i2}\right)$, $P_{i3}=\left(x_{i3},y_{i3}\right)\;$ and $P_{i4}=(x_{i4},y_{i4})$, respectively.

(2) $${Height}_{i}=y_{i1}-y_{i4}$$

(3) $$r_{1}=\frac{1}{N}\sum_{i=1}^{N}\frac{y_{i1}-y_{i2}}{{Height}_{i}}$$

(4) $$r_{2}=\frac{1}{N}\sum_{i=1}^{N}\frac{y_{i2}-y_{i3}}{{Height}_{i}}$$

(5) $$r_{3}=\frac{1}{N}\sum_{i=1}^{N}\frac{y_{i3}-y_{i4}}{{Height}_{i}}$$

**Figure 5:**
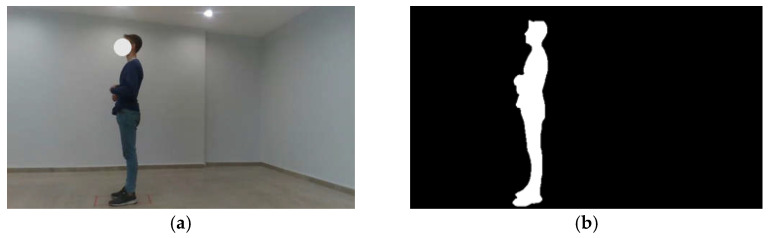
(**a**) Original subject image. (**b**) Binary image extracted using semantic segmentation.

Following the application of semantic segmentation to the original images in the dataset via a CNN model, binary images containing only human and background were obtained. Figure 5 illustrates the original subject image and its binary image, which were extracted using semantic segmentation.

**Figure 6:**
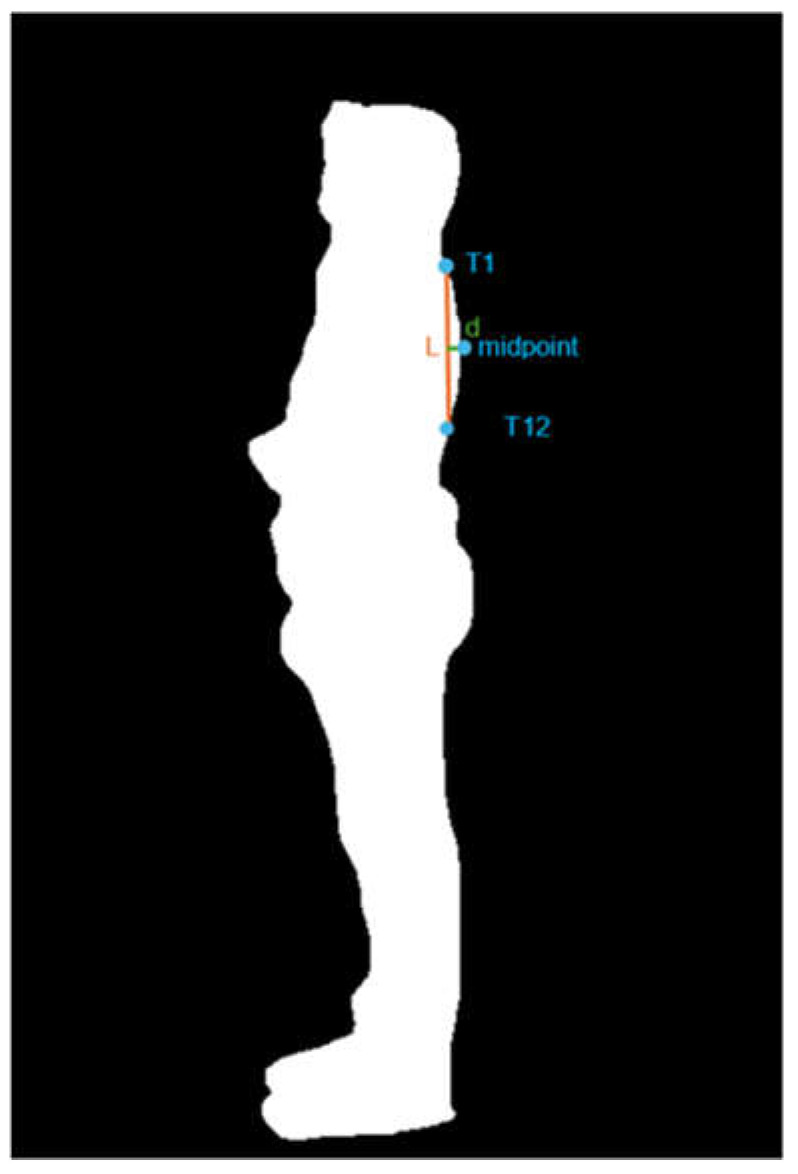
Depicting the lengths *L* and *d* and the T1 and T12 coordinates on the binary image.

Subsequently, the T1 and T12 points were automatically determined using the previously calculated $r_{1}$, $r_{2}$, and $r_{3}$ values. The *L* and *d* parameters were obtained by utilizing the reference points T1 and T12, as illustrated in Figure 6. The kyphosis angle was determined using the formula provided in Equation (1).

### 2.5. Evaluation Metrics

The evaluation of the proposed semantic segmentation algorithm was conducted using a series of metrics, including global accuracy, mean accuracy, mean intersection over union (mean IoU), weighted intersection over union (weighted IoU), and mean boundary F1 score (mean BF score) as given in Table 1 [13,14].

Global accuracy is a metric used to assess the overall accuracy of a model by comparing the number of correctly predicted pixels to the total number of pixels in the dataset. The global accuracy formula is provided in (6) as follows:

(6) $$Global\;Accuracy=\frac{Number\;of\;correctly\;classified\;pixels}{Total\;number\;of\;pixels}$$

In contrast to global accuracy, which assesses the overall proportion of correctly classified pixels, mean accuracy offers a class-specific evaluation by calculating the accuracy for each class individually and subsequently averaging these values. The mean accuracy formula is provided in (7). In this formula, *N* represents the total number of classes.

(7) $$Mean\;Accuracy=\frac{1}{N}\sum_{i=1}^{N}\frac{Number\;of\;correctly\;classified\;pixels\;for\;class\;i}{Total\;number\;of\;pixels\;for\;class\;i}$$

The mean intersection over union (mean IoU) is a frequently utilized metric for the assessment of semantic segmentation models. It offers a comprehensive assessment of the degree of alignment between the predicted segmentation and the ground truth, taking into account both false positives and false negatives. The formulas for intersection over union and mean intersection over union are provided in Equations (8) and (9), respectively.

(8) $${IoU}_{i}=\frac{Area\;of\;intersection}{Area\;of\;union}$$

(9) $$Mean\;IoU=\frac{1}{N}\sum_{i=1}^{N}{IoU}_{i}$$

Weighted intersection over union (weighted IoU) represents a variation of the standard IoU metric that takes into account the relative importance of different classes in a semantic segmentation task. This is particularly advantageous when working with datasets where certain classes are more significant or prevalent than others. The weighted intersection over union formula is provided in (10). In this formula, $\omega_{i}$ represents the weight assigned to class *i*.

(10) $$Weighted\;IoU=\frac{\sum_{i=1}^{N}\omega_{i}\times {IoU}_{i}}{\sum_{i=1}^{n}\omega_{i}}$$

The mean boundary F1 score (mean BF score) is a metric utilized in the field of semantic segmentation to assess the degree of correspondence between the predicted boundaries of segmented objects and the ground truth boundaries. In contrast to metrics such as IoU, which concentrate on pixel-level precision within regions, the mean BF score is specifically designed to assess the accuracy of boundary delineation, which is of paramount importance in tasks where precise object outlines are essential. The formulas for the F1 score and mean BF score are provided in Equations (11) and (12), respectively.

(11) $${F1\;Score}_{i}=\frac{2\times {Precision}_{i}\times {Recall}_{i}}{{Precision}_{i}+{Recall}_{i}}$$

(12) $$Mean\;BF\;Score=\frac{1}{N}\sum_{i=1}^{N}{F1\;Score}_{i}$$

### 2.6. Statistical Analysis

The statistical analysis of the study was conducted using the Statistical Package for the Social Sciences (SPSS) software, version 21.0 (SPSS Inc., Chicago, IL, USA). Prior to conducting parametric analysis, all data were subjected to normality testing (Kolmogorov–Smirnov test) and homogeneity testing (Levene test). Parametric variables were compared using Student’s *t*-test, while nonparametric variables were compared using the Mann–Whitney U test. Descriptive analyses were presented using means and standard deviations for variables with a normal distribution and medians and interquartile ranges (25th to 75th percentile) for variables that were not normally distributed or ordinal. For the purposes of this study, a *p*-value of less than 0.05 was considered statistically significant.

## 3. Results

This study presents an algorithm for the automatic detection of the kyphosis angle. A CNN was trained on the generated dataset to create a segmentation model. The ratios pertaining to the T1 and T12 points were automatically determined on the binary images, and the kyphosis angle was subsequently calculated.

The CNN segmentation algorithm was trained on 440 of the total 553 image data, validated on 56, and tested on 56. Following the segmentation process, the kyphosis angles were calculated using the original images of all subjects in the study for the tests and compared with the gold standard for the evaluation of the algorithm.

The results of the semantic segmentation are presented in Table 1. As indicated in the table, the algorithm exhibits a 99% accuracy rate in the segmentation of person silhouettes.

The preliminary biostatistical evaluation indicated that a sample size of 115 individuals would be required to achieve 85% power and a 0.05% margin of error in the study. A total of 125 volunteers (*n* = 67 female, *n* = 58 male) participated in the proposed study. Subsequent biostatistical analysis revealed that the study was completed with 97.74% power.

Among the 125 participants, 53.6% identified as female and 46.4% as male. All individuals included in the study were healthy young adults, with a mean age of 21.55 ± 1.97 years. The demographic characteristics of the study population are summarized in Table 2.

The results of the automatic kyphosis angle measurement obtained with the algorithm were subjected to statistical evaluation through a comparison with flexicurve measurements. Measurements of 10 patients in the dataset are given in Table 3. In this table, the units of measurement for body length, length, and distance are centimeters (cm), and weight is kilograms (kg). In addition, the actual kyphosis angle given in this table is the angle (°) measured by the expert, while the predicted kyphosis angle (°) is obtained by the proposed algorithm.

The objective of both measurement techniques was to ascertain the clinical applicability and appropriateness of the proposed algorithm as an assessment method. Consequently, the Spearman correlation coefficient and Cronbach’s alpha coefficient were calculated and evaluated. The level of statistical significance for the correlation data was set at an r-value of ≥0.50 and a *p*-value of ≤0.05.

As illustrated in Table 4, the correlation between the kyphosis angle measured by the software and the theta angle obtained from the flexicurve was found to be highly significant (rho = 0.913, *p* ≤ 0.001). In Table 4, the parameters *n* and *p* in Table 4 represent the number of subjects included in the study and the significance level, respectively.

In order to ascertain the internal consistency coefficient, the Spearman correlation was calculated. A test is deemed reliable if its internal consistency coefficient exceeds 0.60, and it is considered to have high internal consistency if its coefficient is above 0.90. The data obtained from the study demonstrated that the current algorithm exhibited high internal consistency, as evidenced by the calculated Cronbach’s α value of 0.947 and the observed kyphosis angle of 31.83 ± 10.05 (Table 5, ICC > 0.95, *p* < 0.05).

## 4. Discussion and Conclusions

The objective of this study was to ascertain the validity of the proposed algorithm in the evaluation of thoracic kyphosis. The results of the study demonstrated that the proposed algorithm, which exhibits high internal consistency and reliability, is a valid method for evaluating thoracic kyphosis.

The evaluation of the flexicurve meter is defined as a non-radiological evaluation method. In approaches that utilize the Cobb angle as a reference, the data obtained from the measurements must be converted into a clinical interpretation through calculations using formulas [10]. However, the proposed posture evaluation algorithm can instantaneously present the requisite evaluation stages regarding posture to the researcher in numerical form within the algorithmic segmentation section. Therefore, the fact that it is straightforward to use in a clinical setting and has been demonstrated to be reliable increases the preference for the proposed posture evaluation algorithm (non-specialist wearing) compared to other non-radiological measurement methods.

A substantial body of evidence exists demonstrating the validity and reliability of the kyphosis angle method with the flexicurve for clinical use. This is evidenced by numerous studies, as referenced in the literature [1,4,10,15,16,17,18]. The kyphosis angle evaluation method with flexicurve is a widely utilized approach in a multitude of studies, including those examining reliability, pain, hyperkyphosis, and postural changes over time. Despite its widespread use in kyphosis angle measurement, the method is subject to criticism, with concerns that the clinical experience and application skills of the practitioner taking the measurement may affect the measurement outcome [4]. In light of the aforementioned details, it is evident that the evaluations were conducted by an experienced physiotherapist, a subject matter expert in the field, with the objective of ensuring the reliability of the flexicurve measurement results. This can be regarded as a noteworthy strength of our study.

A review of the literature reveals a paucity of studies investigating the automatic measurement of kyphosis or Cobb angles. Park et al. [15] constructed a database by acquiring images from 18 subjects with and without specialized outerwear. By employing deep neural networks, the researchers were able to ascertain the thoracic kyphosis and lumbar lordosis angles with an error of less than 3 pixels (0.9 cm). Wong et al. [16] developed an automatic algorithm for the measurement of kyphotic and lordotic angles on 17 radiographic images, employing machine learning techniques. The reported accuracy rates for their method were 95%, 100%, and 100% for T1–T12, T5–T12, and L1–L5, respectively. Galbusera et al. [17] employed a CNN to calculate spinal disorders and deformities in 50 patients from their radiological images. The standard errors of the estimated parameters were reported to range from 2.7° (for the pelvic tilt) to 11.5° (for the L1–L5 lordosis). Alukaev et al. [18] put forth a fully automated deep learning (DL) framework for the vertebral morphometry and Cobb angle measurement derived from three-dimensional (3D) computed tomography (CT) images of the spine obtained from 156 subjects. The results yielded a Pearson’s correlation coefficient of 0.943, 0.928, and 0.996, respectively.

This study recorded the demographic characteristics and non-radiographic images of healthy young adults (mean age: 21.55 ± 1.97 years), followed by the automated assessment of thoracic kyphosis. The evaluation of thoracic kyphosis is conducted by the expert using flexicurve measurements, which are then taken as the gold standard for expert results. Semantic segmentation with a CNN was employed to generate binary images, delineating the body and background components of all subjects. Subsequently, the coordinates of T1 and T12 are identified automatically from the images. Ultimately, the kyphosis angle is calculated using the formula (1). The kyphosis angles of the participants were found to be 31.83 ± 10.05 (*p* ≤ 0.001), exhibiting high consistency (Cronbach’s α = 0.947) and high validity (rho = 0.913, *p* < 0.05).

Table 6 presents a comparative analysis of the findings from the existing literature and the results of the proposed study. As evidenced by the data presented in the table, the number of subjects included in the proposed study and the calculated ICC values are higher than those observed in the existing literature. The range of the intraclass correlation coefficient (ICC) values for the proposed automatic algorithm was found to be 0.925–0.963. The studies presented in the table employed automatic algorithms, whereas the results reported by Grindle et al. [9] were obtained through manual measurements.

The ICC achieved by the proposed method ranged from 0.925 to 0.963, demonstrating excellent reliability and strong agreement with expert-defined reference measurements. In comparison to previous studies summarized in Table 6, Wong et al. [16] reported an ICC of 0.91 using machine learning techniques applied to radiographic images, which is slightly lower than the value obtained in our study, despite relying on high-resolution radiological data. Grindle et al. [9], who performed manual measurements using flexicurve and marker-based methods, reported ICC values between 0.508 and 0.829, indicating moderate to good reliability and higher susceptibility to inter- and intra-observer variability. Alukaev et al. [18] employed a deep learning framework on three-dimensional CT scans and achieved very high Pearson correlation coefficients (up to 0.996 for the Cobb angle); however, their approach depends on radiological imaging, which is costly, invasive, and less suitable for routine use. Galbusera et al. [17] reported an R^2^ value of 0.79 based on CNN-assisted analysis of radiographic images, reflecting moderate agreement with expert assessments, and their dataset was limited to 50 subjects.

Therefore, the proposed algorithm provides a considerable advantage in terms of measurement efficiency. While the conventional flexicurve method requires approximately 15 min per subject due to manual placement, tracing, and calculation steps, the proposed automated algorithm completes the entire kyphosis angle detection process in less than 1 s per subject. This substantial reduction in evaluation time facilitates early diagnosis and significantly reduces the clinical workload associated with manual postural assessments. Moreover, its ability to deliver rapid, objective, and reproducible measurements without the need for radiographic imaging or specialized equipment further enhances its clinical utility and scalability.

The development of an automated decision support system for thoracic kyphosis measurement has the potential to significantly impact clinical practice by reducing reliance on high-cost, radiation-based diagnostic methods and accelerating the measurement process. The demonstrated high intra-class consistency (ICC > 0.95) and internal reliability (Cronbach’s α = 0.947) indicate that this system could provide a reliable and safe alternative for measuring thoracic kyphosis angle. The proposed study may enhance diagnostic efficiency and patient safety, supporting broader applications in clinical settings and contributing to advancements in non-invasive medical imaging technologies.

The contributions of the proposed method can be summarized as follows: The proposed method offers several advantages. Firstly, it employs non-radiological body images, obviating the need for specialized clothing. Secondly, it utilizes an automated algorithm for measuring the kyphosis angle with high accuracy. Thirdly, it avoids continuous exposure of subjects to radiation using non-radiological images. Fourthly, the method is both rapid and cost-effective, due to the absence of specialized clothing.

One limitation of the proposed study is the narrow age range of the participants. A more comprehensive understanding of thoracic kyphosis can be achieved in future studies through the inclusion of participants from a wider age range and the examination of age groups separately.

## Figures and Tables

**Table 1:** Semantic segmentation results.

Global Accuracy	Mean Accuracy	Mean IoU	Weighted IoU	Mean BF Score
0.99879	0.99147	0.98561	0.9976	0.9997

**Table 2:** Demographic characteristics of the participants (n = 125).

Female	Male	Age	Height	Weight	BMI
53.6%	46.4%	21.55	172.77	70.92	23.53
(*n* = 67)	(*n* = 58)	±1.97	±9.91	±18.47	±4.90

**Table 3:** Measurements of 10 patients in the dataset.

Patient Number	Gender	Age	Body Length	Weight	Length (*L*)	Distance (*d*)	Actual Kyphosis Angle	Predicted Kyphosis Angle (θ)
1	Male	22	195	94	32	2.1	29.909	28.070
2	Male	19	185	72	32	1.9	27.088	28.268
3	Male	20	170	65	28.5	1.7	27.212	31.782
4	Male	22	187	96	33	2.2	30.378	26.809
5	Female	21	166	65	28.5	1.5	24.036	23.143
6	Female	20	166	55	27.5	1.7	28.192	25.529
7	Female	18	162	53	25.5	1.3	23.287	24.480
8	Female	21	178	66	27.5	2.1	34.734	31.589
9	Male	23	180	74	33.5	2	27.236	29.960
10	Female	20	173	50	26.5	1.8	30.944	26.330

**Table 4:** Correlations between flexicurve measurements and the calculation results of the algorithm for the kyphosis angle.

Spearman’s Rho (r)	0.913 *
*p*	≤0.001
*n*	125

* Correlation is significant at the 0.01 level (2-tailed).

**Table 5:** Validity of proposed algorithm.

Mean ± SD	ICC (95%)	Cronbach’s α	*p*
31.83 ± 10.05	0.925–0.963	0.947	≤0.001 *

* *p* < 0.05.

**Table 6:** Comparison table with the studies in the literature and the proposed study.

Authors	Method/Dataset	Subject Number	Result
Wong et al. [16]	Machine learning/	17	ICC = 0.91
	radiographic images		
Galbusera et al. [17]	CNN/radiographic images	50	Correlations in variances (R^2^) = 0.79
Alukaev et al. [18]	Deep learning/radiographic images	156	Pearson’s correlation coefficients for the Cobb angle = 0.996, for the vertebral body = 0.943, and for the intervertebral disk = 0.928
Grindle et al. [9]	Manual measuremet with flexicurve and marker	40	ICC = 0.508–0.829
Proposed study	CNN and coordinate measurements/camera images without special wearing	125	ICC = 0.925–0.963

## Data Availability

The data presented in this study are available on request from the corresponding author due to ethical restrictions and the need to protect participant confidentiality.
